# Intergenerational wealth transmission and homeownership in Europe–a comparative perspective

**DOI:** 10.1371/journal.pone.0274647

**Published:** 2022-09-28

**Authors:** Or Cohen Raviv, Thomas Hinz

**Affiliations:** Cluster of Excellence "The Politics of Inequality and The Department of Sociology, University of Konstanz, Konstanz, Germany; Sapienza University of Rome: Universita degli Studi di Roma La Sapienza, ITALY

## Abstract

The literature on social and wealth inequality has long acknowledged the importance of intergenerational wealth transmission (IWT) to inequality in homeownership tenure. However, it has paid insufficient attention to the institutional structures that moderate these inequalities. Therefore, in this study, we ask how institutional factors via differential housing finance systems and governmental housing policies, moderate the association between IWT and homeownership tenure. This is done by using the framework of housing regime configurations and mortgage market financialization. To do so, we pooled data for 20 European countries from the European Central Bank’s Household Finance and Consumption Survey (HFCS) for 2010–2017, for household heads aged 25–40. Our main findings show consistent contradiction to the welfare state–homeownership “trade-off” hypothesis: that is, when the rental market is more heavily regulated, it is actually young adults who benefited from IWT or who received higher value of IWT have a higher probability of mortgaged homeownership. Paradoxically, when housing finance institutions are more active and generous, the wealthiest young adults hold an advantage in mortgaged homeownership. Therefore, liberal mortgage markets actually serve to enable wealthier young adults to reproduce and preserve their parental wealth status. Further, when housing prices are less affordable (median mortgage-to-income ratio), those who have received a higher amount of IWT hold an advantage in mortgaged homeownership. We discuss the implications of our findings, which cut across the socioeconomic, spatial, and demographical arenas.

## Introduction

There is growing interest in the role of parental financial aid on the wealth prospects of their offspring, particularly in relation to achievement of homeownership, since housing assets constitute the single largest share of net worth for households, which tend to increase in value over time [1,2]. This is the case across most advanced economies, especially following their recovery from the Global Financial Crisis [3]. The importance of intergenerational wealth transmission (IWT) from parents to children for the generation of wealth inequality is likely to increase significantly in the coming years, unless governments regulate its extent [4]. The uniqueness of wealth as an indicator of social stratification stems from the fact that it differs from other sources of stratification and inequality, because wealth accumulation is dependent on the history of family wealth accumulation and thus is independent of individual skills and efforts in the labor market or the educational system [5]. One piece of evidence for this is found in the low correlation between household income and wealth across countries, as compared to the high correlation between IWT and wealth [6,7]. Moreover, unlike income, wealth is shielded from adverse life events and acts as a shock absorber during those times [8].

Scholars of social and wealth inequality have long stressed the role played by IWT on the household level in securing access to homeownership, as well as that of wealth accumulation in shaping socioeconomic inequality [9,10]. These studies show that IWT promotes future homeownership or increases wealth prospects in a variety of social contexts, such as in neoliberal countries, like the US [11] and UK [12] and Israel [13] but also in more income-egalitarian countries, such as Sweden [14], Norway [15], and the Netherlands [16]. While these studies considerably advanced our understanding about how the association between IWT and homeownership is stratified across classes and distributed across countries, they provide less focus on the institutional structures that mitigate it at the macro level.

Concurrently, an evolving literature strand of housing financialization and housing regime configurations captures the institutional structures that ease or restrict access to homeownership. That is, it focuses on housing finance systems and government policies that stimulate homeownership or renting across countries [17–22]. However, this body of studies is concerned more about differences between countries in the dynamic between macro-level institutions that shape homeownership tenure than about how these mechanisms contribute to wealth stratification potential for the family unit. A few exceptions address the process of wealth inequality in the housing market among young adults by using different proxies of parental socioeconomic status or household income to predict offspring’s homeownership under different housing configurations, but they do not measure IWT and its extent directly [23–25]. Given the high correlation between IWT, wealth, and homeownership, there is a surprising dearth of studies evaluating the role of parental wealth transmission and its extent when studying the transition to first-time homeownership among young adults under differential institutional arrangements. A recent study documented the importance of IWT in constructing new homeownership typologies for young adults up to current times [26]. However, it did not consider IWT at the household level or the extent of IWT provided, which is a key factor in the process of accessing first-time homeownership. Furthermore, while sorting countries into distinct regimes is helpful for theoretically framing ideological housing structures and making sense of the social world, it also blurs the uniqueness of specific countries’ housing patterns, which vary to some degree throughout history, as well as between different cultures and even housing finance systems. Therefore, the current study builds on the housing-regime configuration literature strand, while also keeping country-specific patterns apparent.

Our study contributes to the sociology debate about wealth and social inequality from the point of view of housing studies. Specifically, we provide a broader perspective from the literature of housing regime configurations and mortgage market financialization, which have been overlooked in previous contributions concerning homeownership and wealth inequality in the sociological literature (also suggested by [7,18,26]). We show how opportunities and constraints are shaped in the housing market and how they vary for the current cohort of young adults, situated under different institutional arrangements at the same point in time. This in turn reveals the complexity of how differential paths to homeownership de- or increase social and wealth inequality within and between different nations in times of intensive growth of wealth inequality and low housing affordability. Further, while many studies have addressed the decline in homeownership among the present cohort of young adults under the frameworks of welfare state retreat, labor market instability, and growing income inequality[27–31], we provide an alternative and more relevant framework centering on the institutions that shape homeownership inequality within and between countries. We also widen the spatial limits in which wealth and homeownership inequality are usually studied by including the region of Central Eastern Europe (CEE), since these countries are commonly viewed as super-homeownership societies. Yet, some countries in CEE have witnessed a trend towards increasing wealth inequality, comparable to the situation in France and Germany (the latter is one of the most unequal countries in the EU [32]), and young adults living in these areas are struggling to access homeownership in the absence of IWT [33,34].

We focus on the present cohort of young adults, as this cohort is struggling with many more barriers in the access to first-time homeownership than previous young adult cohorts, which has been raising critical concern in many Western countries and in the public policy debate about housing affordability. Further, given the wealth potential embedded with homeownership and its central role in the stratification system, surprisingly few studies have investigated the dynamic between macro- and household-level factors in contributing to homeownership and wealth inequality among the young [25,35,36]. The present young adult cohort is facing declining rates of homeownership [37] while living in a time of greater uncertainty and instability than their parents [38]. They live in an era of intensified mortgage market financialization, with more households taking up higher mortgage loans [39,40], increased housing price inflation, growing labor market uncertainty, relatively high unemployment rates [41], income stagnation in many Western countries [42], and shrinking welfare benefits. Hence, a corpus of studies has documented difficulties and delays in transitioning to first-time homeownership across countries as compared to earlier cohorts [38,43]. These circumstances increase the dependence of young adults on IWT and have triggered a shift towards re-familialization, as opposed to the de-familialization that is expected at this life stage [25,44,45].

Against this backdrop, we investigate the following two research questions: first, how the association between IWT and homeownership tenure varies between countries and European regions for the present cohort of young adults; and second, how differential institutional contexts moderate the association between IWT and homeownership tenure. To do so, we provide an up-to-date empirical exploration of the association between IWT and the extent of IWT provided on homeownership tenure within and between 20 European countries, using the Household Finance and Consumption Survey database (HFCS) [46]. We then investigate how this association is moderated by different economic and governmental institutions (using [47–50]). Our main findings show that homeownership is much more equally distributed between young adults situated in different classes in Southern European (SE) and CEE countries, whereas in Western Europe (WE), the quantity of IWT contributes to hierarchical patterns of homeownership, benefitting those from wealthier families of origin. When we added institutional factors to the analysis, we found a consistent contradiction to the welfare state–homeownership “trade-off” hypothesis: that is, when the rental market is more regulated, those who benefited from IWT or who received more IWT have a higher probability of mortgaged homeownership. Paradoxically, when housing finance institutions are more active and generous, the wealthiest young adults hold an advantage in mortgaged homeownership. Therefore, liberal mortgage markets actually serve to enable wealthier young adults to reproduce and preserve their parental wealth status, just as they serve to widen wealth inequality between the older cohort and the present cohort of young adults [38,43]. Further, when housing prices are less affordable (compared to median income), those who have received a higher amount of IWT hold an advantage in mortgaged homeownership. We further discuss the socioeconomic, demographical, and spatial implications of our findings for young adults across countries.

### The significance of homeownership to social and wealth inequality

Homeownership embodies material, social, and psychological advantages that increase the owner’s stake in society and situates households within the broader social order. It provides economic security against periods of strong inflationary trends and the consequent weakening of the “welfare state” [51,52]. One example of this is “asset-based welfare,” where security in old age is based on homeownership (often financed by a home mortgage) rather than a pension [53]. Another example is that young adults in the UK were found to believe that investing in housing assets is the best value-for-money retirement option, due to the neo-liberal policy which significantly reduced old-age pension allocations [54]. Homeownership also has symbolic power as a highly visible representation of status [55]. Owning a more valuable home than average for that age cohort can play an important role in enhancing a young person’s status, by showing they are “ahead” in the accumulation of material goods [56]. Furthermore, homeownership provides higher levels of subjective wellbeing, since it is accompanied by feelings of stability and security, while also providing higher levels of residential and life satisfaction [57,58]. Homeownership (or homeowners) also influences political actors. This is because homeowners often vote to lower levels of public spending and taxation, since they invest and extract wealth from their housing assets, as opposed to renters. This, in turn, to some extent influences government leaders to shape their political ideology accordingly (from both right- and left-wing parties), in order to attract voter support [59,60].

Mortgage loans are the main mechanism driving homeownership, especially for middle- and lower-class young adults with limited resources and support. Such loans are important drivers of social mobility. Thus, differences in access to mortgages and homeownership are fundamental sources of social inequality [13,61]. Access to mortgages also depends heavily on family resources, since IWT enables young adults to become homeowners’ sooner, make larger down payments, and acquire higher-value property [62]. The equity accumulated in housing assets also passes down through the generations, contributing to the persistence of inequality [63]. Thus, IWT stratifies access to homeownership and the prospects for accumulating wealth. Nevertheless, a comparative study found IWT to be a weaker predictor for household wealth than income among the elderly cohort [47]. Similarly, in a study on young adults in the UK, IWT was also found to be less important for household wealth accumulation than other sociodemographic characteristics [64]. It is worth mentioning that homeownership can also create financial risks. Housing prices may decline (or rise) as a result of economic cycles, changes in the attractiveness of a location, and unforeseen external shocks [10]. The global financial crisis of 2008 is a remarkable example of this phenomenon, leading to home depreciation in the US for many lower-class households that had relied on mortgages [48]. The economic turmoil also changed attitudes toward *mortgaged homeownership* as a secure investment for building future wealth among young adults in the US [49]. While homeownership does entail risks of financial loss, over a long period of time, housing assets have led to more wealth accumulation than other forms of financial investment across advanced economies [50].

#### Intergenerational wealth transmission and homeownership across countries

IWT is essential for purchasing housing assets in many countries. Previous studies reveal that homeowning parents or parents who transfer wealth to their children increase the likelihood of the latter’s becoming homeowners themselves, as was found in France [9], the Netherlands [65], and the UK [54]. One remarkable example consists of recent mortgage deals in the UK, which offer mortgages secured using parents’ savings or home equity [54]. The literature also suggests that a socialization process shapes preference towards homeownership earlier in the life cycle, depending on the parents’ homeownership tenure. Children of homeowners have a higher likelihood of becoming homeowners themselves [56,66] and the longer a child spends in their parents’ home, the higher their likelihood of becoming a homeowner [67]. This leads to the persistence of socioeconomic inequality, as the equity accumulated in housing assets is passed down through generations and sustains inequality [58,63].

In different institutional contexts, different kinds of IWT are more common. The kind of IWT at issue in a given case depends on the relationship between the family, the state, and the housing finance system in a particular country. Taken together, these structure the opportunities available for parents to provide specific forms of financial aid and to meet their children’s needs accordingly [45]. In WE countries, the welfare state is relatively generous, the economic security of the elderly and children are seen as the state’s responsibility, and the rental market is fairly regulated [42,43,45,68]. Thus, the financial status of parents is relatively secure, and they are able to provide their children with help in the form of gifts to make down payments on mortgage loans or to deal with adverse life events. This enables young adults to leave the parental home at an earlier stage than young adults’ in Southern European (SE) countries. This process, however, is also connected to culture and norms differences with respect to transition to first-time homeownership of independence versus dependence of children on their parents, between WE and SE countries [69,70]. In SE countries, the mortgage market is relatively inaccessible and public expenditures for family policies are low. These countries also display a legacy of homeownership, while the rental market is tight and expensive, due to a mismatch between supply and demand, exacerbated by stagnating incomes [71,72]. This, in turn, increases the dependency of children on their parents’ resources. Thus, it is more common in SE countries for IWT to take the form of housing assets or space (co-residence).

The housing market in CEE countries has its own unique historical context. Under the communist regime, tenants were mostly dependent on the state for their employment, and income inequality was low [73].The state provided low-quality public housing, of which there was a shortage of supply [74,75]. With the fall of the communist regime and the transition to the free market, tenants were given the opportunity to purchase their homes below market prices. This was a widespread practice, which created high homeownership rates in CEE. However, because mortgage markets remain relatively underdeveloped and the rental market is kept marginal, young adults are mainly dependent on their family resources to provide access to homeownership, as in SE [33,34]. One implication of this situation is that young adults make the transition to first-time homeownership later in life, postponing their family-formation stage and thus decreasing fertility rates in the CEE region [34]. In several CEE countries, housing equity constitutes the largest share of the national aggregate wealth, which is more equally distributed throughout the population [76]. However, another study that merged data on the richest households with survey data, found increasing levels of wealth inequality among the Baltic countries (as measured by the Gini coefficient) comparable with those of Germany and France [32]. This can be explained by the use of different databases, which include data on the wealthiest 1 percent in the CEE. In this context, these developments may change the dynamics between IWT and the extent to which it predicts homeownership.

#### Institutional forces driving homeownership

To investigate the institutional structures that moderate the association between family financial resources and homeownership, we apply an institutional framework originating in the literature of housing studies. Specifically, we draw on the theoretical framework of housing regime configurations in the context of mortgage market financialization. We suggest that the dynamic between those institutional arrangements and the family unit distributes opportunities or constraints for first-time homeownership to a varying extent. Thus, we argue that they also shed light on how the process of wealth inequality is structured at the critical stage of young adulthood within and across countries.

Since the 1980s, the mortgage market has experienced gradual finanacilization, which means that the role of housing has shifted housing assets from being seen as a social right, as part of the social good, and as a source that facilitates access to homeownership under state regulations to a source of advancing wealth accumulation in housing assets per se [39,77]. This change was made possible by an institutional shift towards the increasing liberalization of credit. By the end of the 1980s, most lending constraints had been eliminated in nearly every developed country, and financial legislation opened the credit market to lower-income groups. These new regulations created improved access to credit, contributing to the democratization of credit, which enabled previously excluded segments of the population to participate socially and financially in consumer society by means of credit and loans [78]. The restructuring of the mortgage market together with welfare state reattachment, however, shifted the financial risk involved in mortgage loans to vulnerable social groups, with the remarkable example of the Global Financial Crisis (GFC) in 2008.

In this context of mortgage market finanacilization, a growing body of scholarship on housing regime configurations has developed. It is mainly, but not solely, focused on the role of the housing finance system as the main institution that shapes homeownership across countries, which is absent in the scholarship on welfare state regimes [79–81] and in the literature about labor market instability and growing income inequality [30,31,37].This framework seems ideal for a comparative study of homeownership inequality among young adults, since it captures the penetration of mortgage liberalization across countries that has burdened the present cohort of young adults with higher mortgage debt than previous cohorts of young adults, although to different degrees across countries and classes [26,82,83]. We next draw on the main macro-level institutions that have been addressed in the housing regime literature to better understand how they moderate the association between family financial support and homeownership within and across countries.

Housing finance institutions (including the mortgage market and banks) greatly affect access to homeownership and vary considerably across European countries [18,84,85]. When housing finance systems are liberal and generous, the maximum loan-to-value ratio (i.e., the maximum loan provided by financial institutions relative to the housing price) is relatively high, the down payment needed is low, and access to homeownership is open to the majority of the population [86]. This often results in a more active housing finance system. For example, Northern European (NE) and several WE countries have active and generous housing finance systems. Housing prices are relatively high and parents are less capable of helping young adult children with homeownership, but the housing finance systems are also more developed, thus enabling individuals to achieve homeownership with less family support [87]. One important implication of the mortgage market financialization for this study is that it drove up housing prices, causing house price volatility in several NE and WE countries. This led children to revert to parental financial resources to finance mortgage loans, even among NE countries, which are the most income-egalitarian countries in the West [88]. The latest studies based on housing regimes configurations, however, point to a negative correlation between homeownership rates and mortgage debt per GDP across time and countries, although in certain countries this relationship tended to be positive [21,89].

By contrast, in SE countries, housing finance systems are relatively conservative and strict (less liberal and generous with mortgage loans). Thus, access to homeownership is restricted to those who have savings or receive IWT. The relatively conservative mortgage market in SE countries is connected to the historical and cultural context and to the socioeconomic conditions of countries in this region. After the Second World War, stricter rent controls negatively affected the profitability of the housing market, resulting in a strong decline in private rental housing investment [90]. Lack of demand caused the mortgage markets in these countries to develop slowly, and a culture of family support for achieving homeownership emerged, as state property was passed down through families [87,91]. SE countries are also generally less affluent (with low GDP per capita) and have relatively modest welfare states [80]. Thus, younger generations depend more on family resources. Yet, in the decade prior to the GFC, Spain, for example, experienced a housing construction boom and housing price increases. This in turn benefited the elderly, enabling them to make transfers to their children [92] while the post-GFC period forced a large share of young Spanish adults into the rental sector [72]. Homeownership rates for young adults have declined more strongly in SE countries than in other European countries over the last two decades [36]. Following this theoretical concept, we expect that when housing finance institutions are less active and less generous, the association between IWT and mortgage homeownership will be stronger. We also hypothesize that, in this context, those who have received greater IWT will have a higher probability of mortgaged homeownership.

Kemeny [79,93], developed a distinction between integrated and dual rental markets. Integrated rental systems include countries with corporatist social structures (Austria, Denmark, France, Germany, the Netherlands, Sweden, and Switzerland), where market and social rental housing compete and are subject to similar housing regulations. The costs and benefits of renting are more similar to homeownership, and most of the population is entitled to rental housing subsidies. Additionally, the quality of both private and public rental housing is relatively high, blurring the distinction between public housing and the private rental market [94]. Dual rental systems, by contrast, are characterized by a strict separation between the public and private segments of the rental market. This situation is found mostly in countries with liberal welfare systems but also in Belgium, Finland, Italy, and Norway. In this system, the government subsidizes and promotes homeownership, while the public rental segment is smaller than in integrated rental systems. In dual systems, access to social housing is means-tested and often stigmatized, since the renters living in social housing projects tend to be marginalized groups. Correspondingly, it is characterized by lower quality [94]. Since 1990, there has been greater reliance on market mechanisms in the integrated rental markets, but the distinction between these two systems remains pronounced [95]. In addition, countries with corporatist legacies place greater emphasis on housing as a protected and stable dwelling unit [21], rather than on the financial asset value of housing and its role in building household wealth. Hence, housing policies that heavily regulate and subsidize the rental market make purchasing a home less attractive for tenants. Moreover, the German-speaking countries are characterized by a comparatively protected rental market, along with stricter housing finance systems and fewer incentives for purchasing a home, with the exception of the Netherlands (e.g., Germany imposes high transfer taxes on real estate and offers no mortgage-interest tax deductions for home buying [96]. This in turn leads to lower homeownership rates (e.g., in Germany, Austria, and Switzerland) [97].

In this context, the “trade-off” hypothesis was developed [22,81,98]. It argues that in countries with poor public welfare provision for the elderly, households are forced to make private provision for old age through homeownership, which is a form of investment. In countries dominated by rental housing, by contrast, housing is more likely to be perceived as a social right. However, recent studies show that the “trade-off” hypothesis is no longer valid in current times. They found no correlation, or even a positive one, between the level of government social expenditures and homeownership rates in WE countries [21,88,99]. These new trends contribute to a greater dependency on IWT when it comes to accessing first-time homeownership. Thus, we hypothesize that if governments regulate the rental market more heavily, the association between IWT and homeownership will be stronger than when rental regulations are less restrictive. We also hypothesize that if governments regulate the rental market more heavily, those who received a higher amount of IWT will have a higher probability of homeownership with and without a mortgage. Finally, we adhere to the same hypothesis with regard to government housing policies oriented toward increasing the share of public housing.

The housing literature accounts for other complementary factors that affect the financial dependency of offspring on family resources in accessing homeownership. Variation in housing prices between countries may affect access to homeownership and affordability for young adults as well [66]. In countries with higher housing prices, one would expect young adults to be more dependent on financial support from their parents in accessing homeownership. On the other hand, one must also consider the countries’ general level of economic development (GDP per capita): in richer countries young adults are more likely to be accepted as mortgage holders because of their greater chances of gaining future income [23]. Therefore, one would expect young adults in richer countries to be less dependent on family financial support. Yet, researchers have observed the trend of re-familialization even in Scandinavian countries, which are characterized by the most generous welfare systems [88]. Thus, we hypothesize that when housing prices are higher (i.e., less affordable), the association between IWT and the probability of mortgaged homeownership will be stronger. We also hypothesize that in this context, those who have received greater IWT will have a higher probability of mortgaged homeownership.

### Data, sample, and variables

We drew the data from the three waves of the Household Finance and Consumption Survey (HFCS), namely 2010, 2013–2014, and 2017, which is conducted by the European Central Bank. This survey has been held every four years since 2010 and includes comprehensive information about household wealth and liabilities. For the purpose of the present study, the unit of analysis is the household, because homeownership and mortgage debt are best viewed as a characteristic of households, rather than of individuals [58,100]. The first wave covered 68,627 households, the second wave 84,829 households, and the third wave 84,611 households. The survey includes information for 16–22 European countries in total (depending on the particular wave). Our sample of analysis consists of 20 countries that have a sufficient number of cases for the variables of interest: Austria, Belgium, Cyprus, Germany, Estonia, Spain, Finland, France, Greece, Hungary, Ireland, Italy, Luxemburg, Latvia, the Netherlands, Poland, Portugal, Slovenia, Slovakia, and Lithuania. Unfortunately, the HFCS does not include more Scandinavian countries, except for Finland.

For statistical purposes, the HFCS defined the household representative person on the basis of the UN/Canberra definition. It applies the following criteria in the order listed, until a single appropriate reference person is identified: household type (partners in a de facto or registered marriage, the presence of dependent children, a single parent with children); the person with the highest income; and finally, the eldest person in the household. We included young adults aged 25–40 in our sample. The common age range used for young adults is 25–34 [36,101], but in view of delays to marriage and family formation inherent to the current generation [38], the age range was extended to 40. The total sample size for the three waves is n = 25,946 households.

The analyses in this study are based on the HFCS user guide [102], which specifically addresses the problem of high rates of non-response regarding questions about wealth (net wealth and liabilities). The current study found a high rate of missing values for the independent variable (IWT). In order to solve this problem, the HFCS suggested multiple imputation techniques for estimating the missing values of non-responding households. This estimation of missing observations is conditional upon observed variables that can plausibly explain the pattern of missing information. The number of imputations provided by the HFCS is five [102], which seems to be the generally agreed number of imputations provided with survey data on household wealth. Further, following the use of multiple imputations, we used The Rao-Wu bootstrap variance estimation method [102,103] following the HFCS instructions, with 1,000 replications.

#### Dependent variable

In order to assess homeownership among young adults, we constructed the categorical variable named *housing tenure*, combining information on homeownership and all mortgage debt taken by the household (on the main residence and other housing assets). The variable was sorted into three categories: *non-homeownership* (renters/other form of dwelling); *mortgaged homeownership*; and *outright ownership* (S1 Fig in the supporting information displays the distribution of *housing tenure* across countries).

#### Independent variables

The main independent variable in the analysis is *IWT*, which we consider a dummy variable distinguishing between households receiving wealth transfers and non-receiving households. We combined this variable with the following two questions [2]: First, did the household receive an inheritance or substantial gift (including money or any other financial asset). Second, did the household inherit its main residences or receive it as a gift. The dummy variable takes the value 1 if either or both answers are yes; otherwise, we assigned it the value of 0. We combined both forms of IWT into a single variable, as our main interest is in the combined effect of IWT with institutional context, in influencing chances of homeownership (To make sure results are replicated across countries we also measured the variation in the size of inheritance or substantial gift *per se*, without homeownership transfer (see supporting information S2 and S3 Figs. The results were mostly replicated, with the exception for Greece for IWT and homeownership tenure and Greece Portugal and Estonia for IWT quintiles and homeownership tenure). We acknowledge that inheritance in the form of gifts or housing assets can also be transferred in a later stage of life and not only at the stage of young adulthood. However, since the HFCS latest wave was launched in 2017, we cannot follow young adults at a later stage.

In order to evaluate the amount of IWT provided (i.e., the quantities of gifts, money, and asset worth), we used a cumulative distribution function of IWT for each country, meaning that we grouped the quantitative values of IWT into quintiles for each country. This practice allows us to compare countries with different purchasing power parity (PPP). We named this variable *IWT quintiles*, and it includes only households that have received *IWT*. Only 14 countries in our sample had a sufficient number of observations on this variable.

We also controlled a series of household economic and socio-demographic variables, which were found to be correlated with homeownership [61,104]; the *household income* (non-asset income) variable refers to the gross annual income from all resources (income from work/self-employment, rental income from real estate, financial investments, social security, and other resources. Information on net income is not available in the data). Here again, we generated income quintiles for each country. This relative definition of income makes it possible to compare those in similar (relative) class positions across countries with different PPP. *Academic education* is based on the highest ISCED level attained (International Standard Classification of Education, 2011) and grouped into three categories: lower than high school graduation, high school graduation (and vocational education), and academic education. *Age* is a continuous variable indicating the household representative’s age in years. *Household size* is also a continuous variable indicating the number of individuals in the household (the last two variables were centered on the mean). *Marital status* is a categorical variable with three categories, for which a value of 1 denotes “married/consensual union on a legal basis,” a value of 2 denotes “single,” and a value of 3 denotes “divorced, separated or widowed”—in the case of young adult households, the percentages in the last category were very low (Married 43%; single 49%; divorced, separated, and widowed 8%). *Employment status* is a dummy variable for which 1 denotes “employed household representatives” and 0 denotes “unemployed/not in the labor force” (the sample household level characteristics are exhibited in Table 1).

**Table 1:** Descriptive statistics of the sample household level characteristics.

Variable	Obs	Mean	Std. Dev.	Min	Max
***Housing tenure***; non HO	25945	.44	.5	0	1
mortgaged HO	25945	.36	.48	0	1
outright HO	25945	.2	.4	0	1
***IWT***	25945	.24	.43	0	1
***IWT quintiles***	3946	2.95	1.42	1	5
***Household income quintiles***	23253	3	1.41	1	5
***Education level***; elementary school	25881	.13	.34	0	1
secondary education	25881	.43	.49	0	1
Tertiary education	25881	.44	.5	0	1
***Marital status***; single	25941	.49	.5	0	1
Married	25941	.43	.5	0	1
Divorced/separated/widowed	25941	.08	.27	0	1
***Employment status***	25910	.87	.33	0	1
Age (centered)*	25946	0	3.95	-6	3.97
Household size (centered)*	25946	0	1.41	-1.5	11.46

*Age and *Household size* were centered around the mean.

In order to identify the extent to which institutional and structural factors moderate the relationship between *IWT* and *IWT quintiles* and homeownership, we used the following five macro-level variables. In order to match these to the timeline of the HFCS survey waves, we drew data for the macro indicators from 2010–2017 (or earlier whenever possible, since some household received *IWT* before the first wave of the HFCS). In order to capture the level of activeness and accessibility/generosity of housing finance institutions, we used two measures that allow between-country comparisons: *Total outstanding residential loans-to-GDP ratio* reflects the mortgage market’s activeness in a given country [105]. Higher residential loans-to-GDP ratios indicate countries where homeownership is mainly achieved through mortgage loans, while lower levels indicate alternative means of attaining homeownership (e.g., *IWT*). *Max LTV ratio* is the second macro variable capturing access to mortgage (list of references under Table 1 below). It represents the level of generosity of the housing finance system: A higher *max LTV* ratio means that lower-class households are able to access the mortgage market and reflects the level of liberalization and risk that housing finance institutions are willing to absorb in the event of a default [106]. Although these two variables are similar, the latter relates to mortgage finance conditions, while the former indicates the final mortgage market results (for example, the same country can have a relatively high max LTV ratio and a relatively low residential loans-to-GDP ratio).

In order to capture governmental housing policies oriented towards increasing the share of renters, we used two indicators. The first is *rent control level*. This is a quantitative variable that reflects the extent to which the government regulates the initial rent levels and the ongoing rent increase landlords can demand from tenants (ranked 1–5 by the OECD [107]). Higher levels in this dimension mean higher rent controls imposed by the state; lower levels mean the initial rents and ongoing rents are freely negotiated between landlord and tenant. We also measured the share of *public housing stock*, by calculating the average share of housing stock from the total number of dwellings in a given country, for two available time points (around 2010 and 2020) [108].

As a means of accounting for housing affordability, the literature suggests measuring, on the one hand, a purchase-related variable, such as house prices or mortgage loans, and, on the other, a financial capacity indicator, such as household income [109,110]. Accordingly, using the HFCS database, we self-calculated the median *mortgage-to-income ratio* for each country (median mortgage debt divided by the median income for each country for the three survey waves) which makes housing affordability a comparable measurement between countries with different PPP. A higher *mortgage-to-income ratio* means less affordable housing. We also controlled for country-level affluence using country dummies, GDP *per capita* [111,112] and generated a dummy variable indicating post-communist countries (followed by [104]). The descriptive statistics of the macro indicators are shown in Table 2.

**Table 2:** Descriptive statistics of macro-level indicators, by country.

Region	Country	Residential loan to GDP%	Max LTV%	Public housing stock%	Rent control level	Mortgage to income%
WE	Austria	27	82	23.80	2.5	23
	Belgium	48	83	4.23	1.5	26
	Germany	43.55	75	3.18	3.5	25
	Finland	41.53	90	11.89	0.7	17
	France	39.39	91	13.77	2	31
	Ireland	53.49	88	12.71	1.2	26
	Luxembourg	48.77	91	1.58	2.3	23
	Netherlands	101.23	105	36	3.9	22
SE	Greece	36.22	74	n.a.	1.7	30
	Italy	32.22	73	4.20	1.5	28
	Portugal	59.16	81	2.02	2	21
	Spain	53.21	71	1.13	1.5	24
	Cyprus	60.41	80	n.a.	n.a.	34
CEE	Estonia	34.3	85	1.10	n.a.	16
	Hungary	18.15	77	2.82	1.67	17
	Latvia	25.09	90	1.89	n.a.	16
	Lithuania	25.09	85	0.81	n.a.	13
	Poland	19.05	90	8.86	1	19
	Slovenia	13.26	73	5.02	0.67	20
	Slovakia	20.54	95	1.62	n.a.	21

Note.

Average residential loan to GDP% for years: 2008–2017 [105].

Average Typical LTV for 2007 [18,113].

Max LTV 2011/2015 [114].

Max LTV 2017 [115].

Max LTV for Luxemburg [114].

Max LTV Spain 2008 https://www.housing-finance-network.org/index.php?id = 348.

Rent control level (only available for 2009) [116].

Average GDP per head of population for years: 2009–2017 [111].

Average GDP per capita for Cyprus (2009–2017) [112].

Average Share of public housing stock (2010, and 2020) [108].

### Findings

The finding section is organized accordingly; we first introduce the findings of the descriptive statistics and multivariate analyses to investigate the association between IWT and IWT quintiles and homeownership tenure, within and across countries. In the second part we will add the institutional factors that moderate this association.

### Descriptive statistics for housing tenure by IWT and IWT quintiles

**Fig 1:**
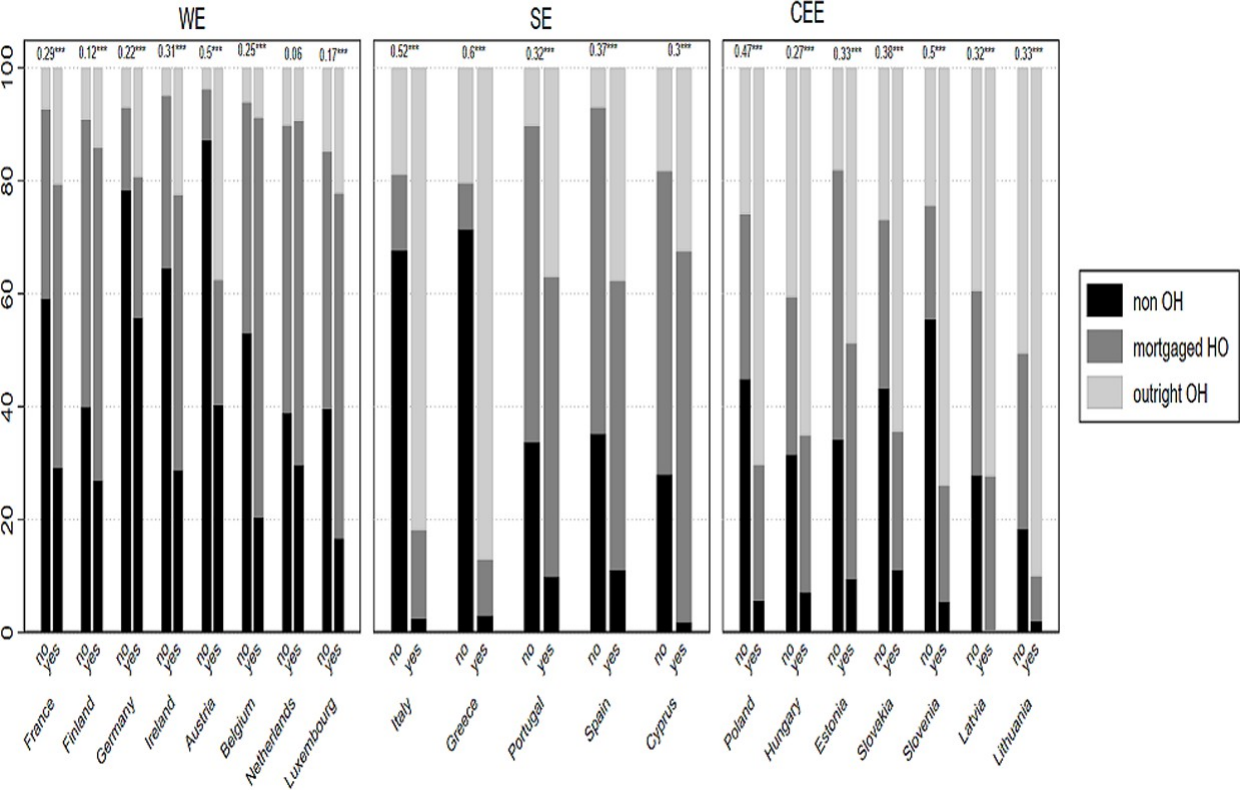
*Housing tenure* categories by *IWT* and country. Note. Outside the Bars; Cramer’s V and P values *** p<0.05, ** p<0.01, * p<0.001, Countries are placed in descending order.

We calculated a series of descriptive statistics in order to examine how *IWT*, and the extent of *IWT*, is associated with *homeownership tenure*, for each country. Fig 1 shows the rates of *housing tenure* categories by *IWT* for each country.

Fig 1 reveals a stratified pattern of homeownership categories that are dependent on *IWT* across countries. In most countries, households benefitting from *IWT* have higher likelihoods of *outright ownership* as compared to non-beneficiaries of *IWT*. In particular, in CEE countries, *IWT* beneficiaries have higher shares of *outright ownership* (e.g., Lithuania 90%, Slovenia 74%, Latvia 72%, Poland 70%, Hungary 65%), which is also true of SE countries such as Italy (82%) and Greece (87%), as compared to *IWT* beneficiaries in WE countries, including Finland (e.g., Ireland 23%, France 20%, Luxembourg 22%, Finland 14%, the Netherlands 10%). The stronger correlation between *IWT* and *outright ownership* among CEE and SE countries as compared to WE countries (with the exception of Austria), is also confirmed by the higher Cramer’s V values for the former. By contrast, *IWT* beneficiaries situated in WE countries are more likely to have *mortgaged ownership*, with at least half of the *IWT* beneficiaries in those countries having *mortgaged homeownership*, such as Belgium (71%), Luxemburg (61%), Finland (59%), and Ireland (49%). The high prevalence of renting (*non-homeownership*) among German-speaking countries (Germany, Austria, and the Netherlands) can be explained by state regulations supporting the rental sector as an alternative to homeownership [18]. As for non-*IWT* recipients, the lack of family financial support pushes them into the rental sector. Yet, among CEE countries, the latter have relatively high share of *outright ownership* independent of *IWT* (more than 40% of *outright ownership* for non-*IWT* recipients in Hungary, Lithuania, and Latvia).

**Fig 2:**
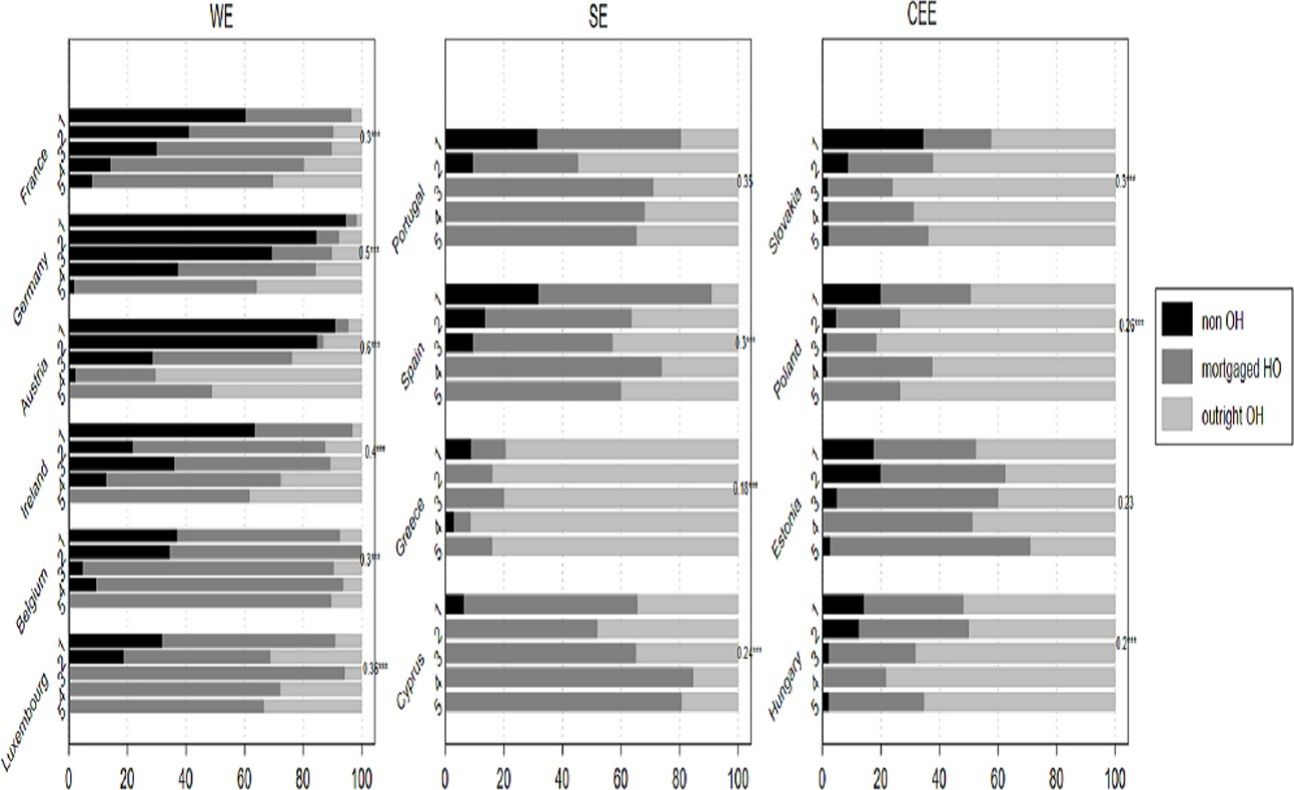
Housing tenure categories by quintiles of the amount of IWT and country.

We are also interested in how different levels of *IWT* contribute to homeownership, in different institutional context. Fig 2 below shows the rates of homeownership categories by *IWT quintiles* for each country (only for *IWT* beneficiaries).

Fig 2 reveals three main findings: First, across most countries, individuals situated in the highest *IWT quintiles* are more likely to have *outright ownership*, while those situated in the lowest *IWT quintiles* are more likely to be renters. Second, hierarchal patterns of homeownership are clearly more evident among WE countries (e.g., Austria, Germany, France, and Ireland), as those in the highest *IWT quintiles* are more likely to have *outright ownership* or *mortgaged homeownership* as opposed to those in the lower *IWT quintiles*. These stratified patterns are substantially less pronounced among CEE countries, with a large share of those situated in the lower *IWT quintiles* having *outright ownership*. The stronger correlation between the extent of *IWT* and *outright ownership* or *mortgaged homeownership* among WE countries compared to the CEE countries, is also confirmed by the higher Cramer’s V values for the former. This is an indication that housing assets are more equally distributed among CEE countries and in SE countries to some degree (except for Portugal). Third, among WE countries, Spain, and Portugal, households situated in the highest *IWT quintiles* tend to have *mortgaged homeownership* rather than *outright ownership*. This is expected, as the latter countries are characterized by more developed and generous housing finance systems (as opposed to in CEE), coupled with unaffordable housing prices, which cause young adults to revert to using the *IWT* at their disposal by taking up mortgaged loans to purchase housing assets. Thus, among the latter, those endowed with higher-value of *IWT* are at an advantage when it comes to *mortgaged homeownership*.

#### Country-specific multivariate analysis

**Fig 3:**
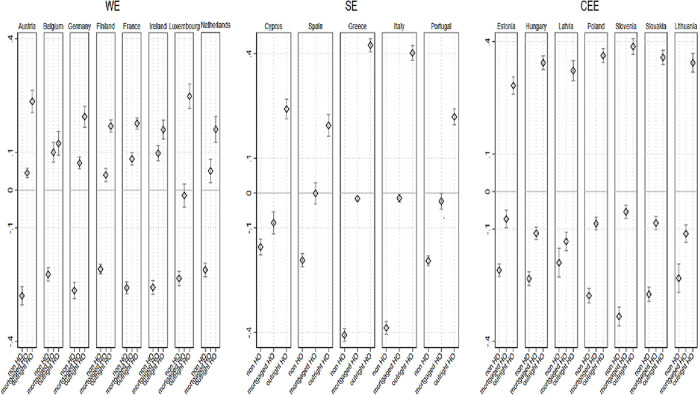
AME for pooled multinomial logistic regression predicting the difference in probability of housing tenure, by IWT and control variables.

These descriptive statistics already produce interesting findings, but they do not reveal the extent to which *IWT* contributes to *homeownership tenure*, independently of other household characteristics, both between and within countries. Since our sample includes 20 countries, we are not able to perform a multilevel analysis to investigate country-level effects with reliable variance estimates [117]. Thus, we pooled cross-sectional data and calculated pooled multinomial logistic regression models to estimate the probability of being in a particular category of *housing tenure* by *IWT*, controlling for household economic and socio-demographic attributes, with a set of country-level fixed effects: country #year dummies, GDP per capita, and post-communist countries dummy (S1 Table in the supportive information the Relative Risk Ratios from a pooled multinomial logistic regression, which predicts the difference in the probability of *homeownership tenure* with control variables). In order to facilitate the interpretation of the data, we display in Fig 3 below the average marginal effects (AME) for these models. The AME reflect the percentage-point difference in the dependent variable (*housing tenure*) associated with a one-unit change in the independent categorical variable, net of control variables at their observed values [118]. An important advantage of AME is that it can be compared across countries.

Fig 3 shows considerable similarities across countries in the association between *IWT* and homeownership categories. In all countries, the probability of *outright ownership* is statistically significant and higher for *IWT* beneficiaries than for non-beneficiaries, net of control variables. Yet, the gap in *outright ownership* between these parallel groups is larger among CEE countries, as well as in Greece and Italy (e.g., Greece: 43 percentage points; Italy: 40 percentage points; Slovenia: 39 percentage points; Poland: 36 percentage points; Lithuania: 34 percentage points), than it is among WE countries (e.g., Belgium: 12 percentage points; Ireland, the Netherlands, and Finland: 16 percentage points; France: 17 percentage points). This is because the former countries are characterized by comparatively less developed housing finance institutions and family financial support is a social norm, which in turn strengthens the association between *IWT* and *outright ownership* to a greater degree than in other countries. Additionally, in all the countries, the probability of *non-homeownership* is statistically significant and lower for *IWT* beneficiaries than for non-beneficiaries, net of control variables. Indeed, the data provides strong evidence that access to homeownership is produced through the accumulation of family resources over generations.

We also expected that young adults living in countries characterized by less affordable housing prices (higher mortgage to income ratio) will be more dependent on family financial resources; hence, we expected the relationship between *IWT* and *mortgaged homeownership* to be stronger in this context. Indeed, we found that *IWT* beneficiaries have a statistically significant and higher probability of *mortgaged homeownership*, compared to their peers, and that this association is stronger in WE counties with less affordable housing, holding all other variables constant (e.g., Belgium: 13 percentage points; Ireland: 12 percentage points; the Netherlands: 7 percentage points; France: 6 percentage points). By contrast, in CEE countries, we found a statistically significant and negative association between *IWT* and *mortgaged homeownership*. In the latter countries, *IWT* beneficiaries have a lower probability of *mortgaged homeownership* than non-beneficiaries (e.g., Latvia: -12 percentage points; Lithuania and Hungary: -10 percentage points; Estonia: -6 percentage points). This means that those have not benefited from *IWT* in CEE countries tend to have *mortgaged ownership*, while *IWT* beneficiaries tend to have *outright ownership*. Among SE countries, the association between *IWT* and *mortgaged homeownership* is negative and statistically insignificant.

**Fig 4:**
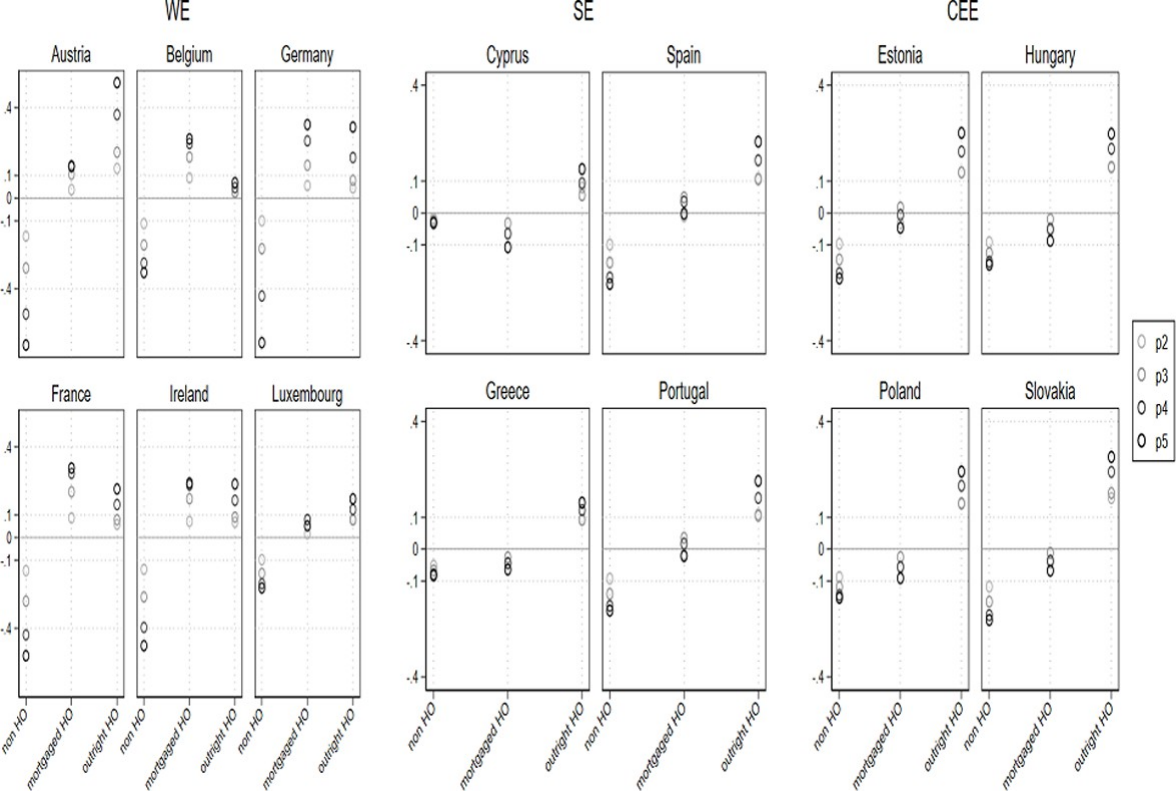
AME for pooled multinomial logistic regression predicting the difference in probability of housing tenure, by IWT quintiles and control variables.

We turn now to investigate the association between the extent of *IWT* and *housing tenure*, in different institutional contexts. In order to do so, we ran the same equations detailed above, but on *IWT quintiles* (S2 Table in the supportive information). Fig 4 displays the AME for the pooled multinomial logistic regression, which predicts the difference in probability of being in any of the categories of *housing tenure* by *IWT quintiles*, with control variables.

Fig 4 shows that the stratified patterns of *housing tenure* found earlier among Western countries hold, net of control variables. The Fig reveals that across countries, households that are situated in the highest *IWT quintiles* have a higher probability of *outright ownership* and a lower probability of *non-homeownership* than those from the lower *IWT quintiles*, net of control variables. The Fig also clearly shows a wider *IWT* class-based gap in *housing tenure* among WE countries, and in particular among German-speaking countries. Those situated in the highest *IWT* quintile in Germany and Austria have a 30 and 50 percentage point higher probability of *outright ownership* and a 36 and 16 percentage point higher probability of *mortgaged homeownership* than those situated in the lowest *IWT* quintile. By comparison, in Spain, Portugal, and France, households situated in highest *IWT* quintile have around a 20 percentage point higher probability of *outright ownership*, with Belgium and France having a 29 percentage point higher probability of *mortgaged homeownership* and Ireland having a 26 percentage point gap, as compared to households situated in the lowest *IWT* quintile. Among CEE countries, *IWT* class-based gap in *outright ownership* is also relatively wide, as those from the highest *IWT* quintile have a 20–28 percentage point higher probability of *outright ownership*, compared to those situated in the lowest *IWT* quintile (e.g. among Slovakia, Hungary, Estonia and Poland). Yet, among CEE and SE countries, the class-based gap in *mortgaged homeownership* is considerably narrower, as those from the highest *IWT* quintile have the same or even a lower probability of *mortgaged homeownership* than those situated in the lowest *IWT* quintile. This means that the distribution of housing assets is more equal among young adults of different means in CEE and SE countries. Hence, in this context, households with fewer means have better wealth prospects as they are more likely to hold housing assets through mortgages than their parallel in the lower quintiles among WE countries.

### Introducing country-level indicators

Our main research aim is to investigate whether and to what extent economic and political institutions moderate the association between family financial resources (*IWT*) and homeownership. First, we expected a “trade-off” between micro- and macro-level institutions: When housing finance institutions are less active and generous (as measured by *residential loan-to-GDP ratio* and *max LTV ratio* respectively), the association between *IWT* and *mortgaged homeownership* will be relatively stronger. We hypothesized the same with regard to the extent of *IWT*. Second, because countries characterized by higher levels of rent control are less oriented towards increasing the share of homeowners, we expect children to be more dependent on family financial resources. Thus, we expected that when governmental regulations support and protect the rental market (as measured by *rent control level* and *share of public housing*), the association between *IWT* and *mortgaged homeownership* or *outright ownership* will be relatively stronger than when rental regulations are less pronounced. We hypothesized the same with regard to the extent of *IWT* provided in this context. Third, we hypothesized that when housing assets are less affordable (as measured by median *mortgage-to-income ratio)* the association between *IWT* and *mortgaged homeownership* will be relatively stronger than when housing assets are more affordable. We hypothesized the same with regard to the association between the extent of *IWT* provided and *mortgaged homeownership*.

In order to test these hypotheses, we introduced country-level indicators and their interaction terms with *IWT* to pooled multinomial logistic regression equations, in order to estimate the extent to which these country characteristics moderate the relationship between *IWT* and *housing tenure*. Because models with many cross-level interactions tend to become over-fitted and not estimable, we proceeded step-by-step and only integrated a single interaction at a time into the models. Subsequently, each regression equation predicts *housing tenure* as a function of all household characteristics (as first-level variables), plus only one country-level characteristic (as a second-level variable) at a time, with the set of country-level fixed effect: survey year, GDP per capita, and post-communist countries dummy. In the following models, all macro variables were centered around the mean. First, we created an empty model (not displayed in S3 Table, see supportive information) including country dummies only and showed that the intra-class correlation is 0.11, which means that 11% of the variation in *housing tenure* is explained by the country of residence. We then created the first model, introduced in S3 Table, which included *IWT*, with *residential loan-to-GDP ratio* and a cross-level interaction between *residential loan-to-GDP ratio* and *IWT*, the set of household socio-demographic characteristics, and the set of country-level fixed effects (country dummies were not included due to perfect multi-collinearity). For models 2–5, we ran the same equation, with a different macro-level variable each time.

**Fig 5:**
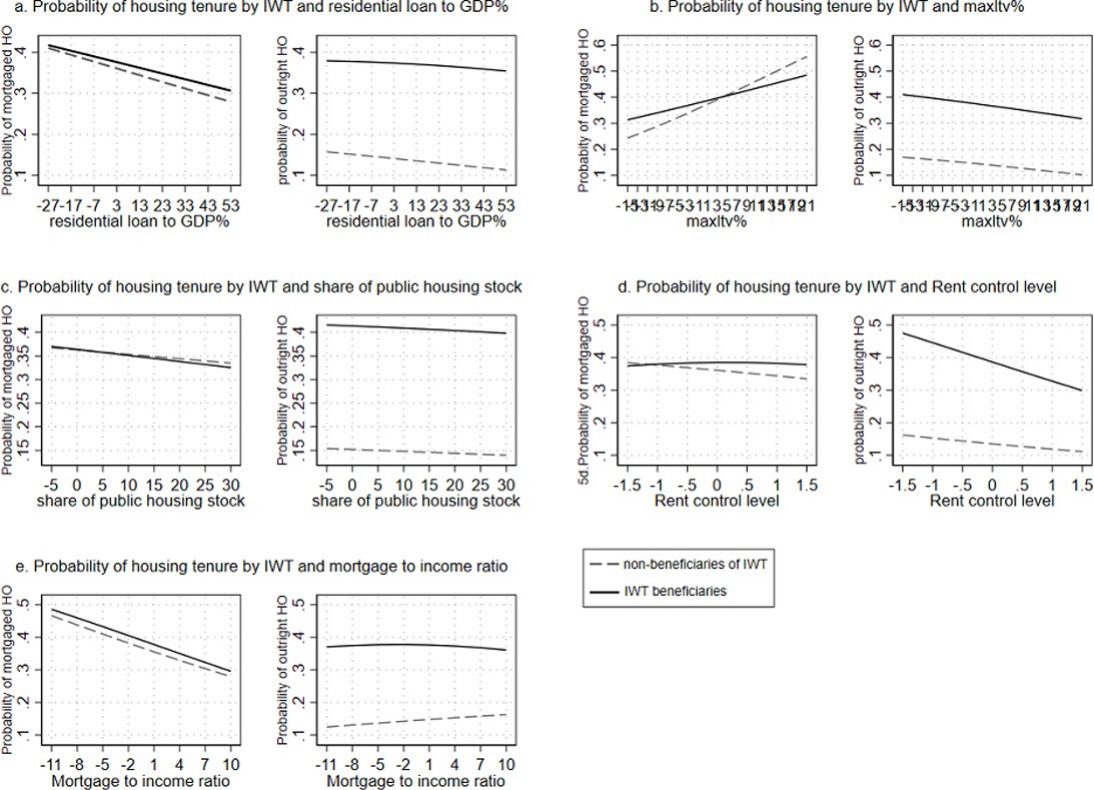
a-e. Predictive margins of pooled multinomial logistic regression predicting the difference in probability of *housing tenure* by *IWT* and macro variables (reference group: *Non-homeownership)*. Note: Macro variables are centered around the mean.

In order to simplify the interpretation of results and because our main research interest is to predict homeownership by cross-level interaction between *IWT* and different macro-level variables, we present the findings for Models 1–5 in Fig 5A–5E. The Figs display the predictive margins of the pooled multinomial logistic regression models, which predict the probability of *housing tenure* by *IWT* and country-level variables. In the Figs, the solid line represents *IWT* beneficiaries, while the dashed line represents *IWT* non-beneficiaries.

When we zoom out to examine all the Figs, we observe a significant main effect for *IWT* in predicting the probability of *outright ownership*, but not necessarily of *mortgaged homeownership*. Thus, overall, *IWT* beneficiaries have a higher probability of *outright ownership*, as compared to non-beneficiaries. We continue with Fig 5A and 5B, which capture the role of housing finance systems in moderating the association between *IWT* and *housing tenure*. Both sides of Fig 5A display a statistically insignificant interaction between *residential loan-to-GDP* and *IWT*, when it comes to predicting *housing tenure* categories. Fig 5B, however, reveals a statistically significant, negative interaction between *max LTV* and *IWT*, as *IWT* beneficiaries have higher probability of *mortgaged homeownership* on the lower levels of *max LTV ratio* than non-beneficiaries (7 percentage points). By contrast, non-beneficiaries of *IWT* have a higher probability of *mortgaged homeownership* on the higher levels of *max LTV ratio* (again 7 percentage points on the other direction). This finding confirms our hypothesis regarding the trade-off between *IWT* and housing finance systems. This means that when housing finance systems are conservative (less generous), those receiving family financial support have higher probability of *mortgaged homeownership* than those who do not. It also shows that when housing finance systems are more generous, the role of family resources makes a somewhat weaker contribution to *mortgaged homeownership*.

Next, we examine the role of governmental regulations in the housing market in moderating the association between *IWT* and *housing tenure*, in Fig 5C and 5D. Both sides of Fig 5C show an insignificant association between the share of *public housing stock* and the probability of *mortgaged homeownership* or *outright ownership*. However, the left-hand panel in Fig 5D reveals a statistically significant, negative interaction between *IWT* and *rent control level* for the probability of *mortgaged homeownership*. While the probability of *mortgaged homeownership* for *IWT* beneficiaries increases somewhat across the levels of *rent control*, the probability of *mortgaged homeownership* for non-beneficiaries decreases as the level of *rent control* increases (i.e., there is an 8 percentage point gap between the groups at the highest *rent control level*). The right-hand side of Fig 5D also reveals a significant interaction effect between *IWT* and *rent control level*, with an advantage for *IWT* beneficiaries in predicting the probability of *outright ownership* across all rent control levels. Yet, the gap between the two groups narrows as the level of rent control increases. These findings are partially in line with our hypothesis, since in the context of a highly controlled rental market, *IWT* beneficiaries hold an advantage in *mortgaged homeownership* and *outright ownership* over non-beneficiaries. Yet, we have not found any evidence for this when the share of *public housing* is introduced into the model.

We were also interested in the way in which housing affordability interacts with *IWT* for predicting *housing tenure* (keep in mind that higher *mortgage-to-income ratio* means that housing affordability is lower). The left-hand side of Fig 5E reveals a statistically significant negative interaction between *mortgage-to-income ratio* and *IWT* when predicting *mortgaged homeownership*. Thus, in line with our expectations, *IWT* beneficiaries have higher probability of *mortgaged homeownership* on the higher levels of *mortgage-to-income ratio*, as compared to non-beneficiaries. Yet, the gap between those two groups is relatively small across the units of *mortgage-to-income ratio*. Thus, the findings support our hypothesis regarding the stronger dependency of offspring on family financial support when purchasing a home, in the context of less affordable housing prices (a higher burden of *mortgage-to-income ratio)*.

**Fig 6:**
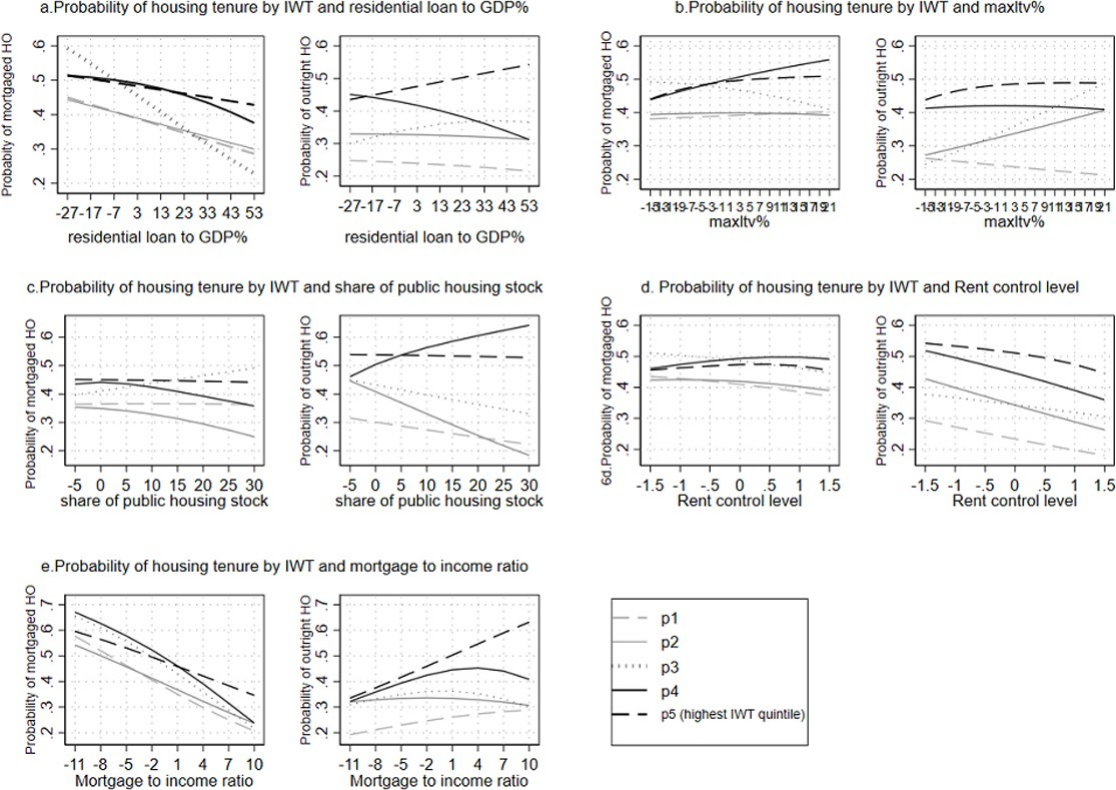
a–e. Predictive margins of pooled multinomial logistic regression predicting the difference in probability of *housing tenure* by *IWT quintiles* and macro variables (reference group: *Non-homeownership)*. Note: Macro variables are centered around the mean.

Our second research aim was to investigate whether and to what extent the value of *IWT* received influences access to homeownership among offspring, while taking institutional factors into account. Thus, we subsequently ran the same equations described above, but this time with *IWT quintiles*. Once again, all macro variables were centered around the mean. Fig 6A–6E below presents the predictive margins of the pooled multinomial logistic regression models predicting the probability of *housing tenure* by *IWT quintiles* and country-level variables (S4 Table in the supportive information).

The Figures in the first row (6a and 6b) capture the role of housing finance systems in moderating the association between *IWT quintiles* and *housing tenure*. We expected that when housing finance institutions are *less* active or *less* generous, the association of *IWT quintiles* and *mortgaged homeownership* will be stronger. Fig 6A reveals a statistically significant, negative cross-level interaction between *IWT quintiles* and residential loan to GDP% for predicting the probability of *mortgaged homeownership*. Surprisingly, the middle class (p3) has the highest probability of *mortgaged homeownership* at the lower levels of residential loan to GDP% and the lowest probability at the highest levels of *residential loan to GDP%*. In contrast, the highest quintile (p5) has the highest probability of *mortgaged homeownership* on the higher levels of *residential loan to GDP%*, compared to the third *IWT* quintile (20 percentage points). The right side of Fig 6A shows a significant, negative interaction effect between *IWT* and residential loan to GDP% when it comes to predicting the probability of *outright ownership*. The gap between the *IWT quintiles* increases at the higher levels of *residential loan to GDP%*: For the highest *IWT* quintile (p5), the probability of *outright ownership* is 33 percentage points higher than for the lowest *IWT* quintile, at the highest level of residential loan to GDP%. Fig 6B exposes statistically significant and positive interaction between *IWT quintiles* and *max LTV* ratio in predicting the probability of *mortgaged homeownership*. Again, at the higher levels of *max LTV* ratio, the probability of *mortgaged homeownership* is the highest for the fifth and fourth *IWT quintiles* (10 percentage points higher), as compared to the lowest *IWT* quintile. Hence, contrary to our expectations, the upper *IWT quintiles* actually hold an advantage in *mortgaged homeownership* when housing finance institutions are *more* active and *more* generous.

The Figs in the second row (Fig 6C and 6D) capture how governmental practices in the housing market moderate the association between *IWT quintiles* and *housing tenure*. We expected that when the rental market is more highly regulated, the association between *IWT* and *mortgaged homeownership* or *outright ownership* will be stronger. The findings presented in Fig 6C and 6D on the whole confirm our expectations. The left-hand side of Fig 6C shows a significant negative interaction effect between *IWT quintiles* and *public housing stock%* for the probability of *mortgaged homeownership*. At the lower levels of *public housing stock%*, the gap between *IWT quintiles* is relatively narrow, increasing at the higher levels of *public housing stock%*. For example, the highest *IWT* quintile probability of having *mortgaged homeownership* is 8 percentage points higher than the lowest *IWT* quintile, at the highest levels of *public housing stock%*. The right-hand side of Fig 6C shows the advantage of the highest *IWT quintiles*, compared to the lowest *IWT* quintile, on the higher levels of *public housing stock%* as well (30 percentage points). In line with these findings, both sides of Fig 6D reveal a significant, negative interaction effect between *IWT quintiles* and *rent control levels*. Thus, we once again observe an advantage for the highest *IWT quintiles* at the higher levels of rent controls as compared to the lowest *IWT* quintile, for predicting *mortgaged homeownership* or *outright ownership*. Hence, the results mostly support our hypothesis that when the rental market is highly regulated (either by higher level of rent control *or* by a larger share of public housing), recipients of greater *IWT* have a higher probability of *mortgaged homeownership or outright ownership*.

We were also concerned with the way in which the extent of housing affordability moderates the association between *IWT quintiles* and *mortgaged homeownership*. We expected that recipients of greater *IWT* will be more likely to have *mortgaged homeownership* when housing prices are less affordable (higher values of *mortgage-to-income ratio*). The left-hand side of Fig 6E does indeed support our hypothesis, since the gap between the highest and lowest *IWT quintiles* in the predicted probability of *mortgaged homeownership* is 14 percentage points higher, at the higher levels of *mortgage-to-income ratio*.

We will now proceed to a detailed discussion and conclusions of the findings in the next section.

## Discussion and conclusion

In this study, we contribute to the sociological literature on homeownership and wealth inequality by shifting the focus towards macro-level institutional explanations originating in housing studies, which suggest how these inequalities can be mitigated. We show how the dynamic between macro-level institutions and family materialistic resources contributes to inequality in access to first-time homeownership for young European adults at the current time. In doing so, we hope to have broadened the sociological literature and debate about homeownership and wealth inequalities to account for theoretical explanations centered around variation in housing finance systems and for the contribution of government programs to homeownership inequality and thereby to wealth inequality within and between countries (also suggested by [7]). We believe that this alternative framework provides broader, updated, and more relevant theoretical explanations than previous frameworks about individual circumstance of accumulative advantage [5,10,63] or other macro-level explanations of homeownership inequality focusing on welfare state retreat, labor market instability, and growing income inequality [28–30].

Our key findings have several implications that cut across socioeconomic, spatial, and demographical arenas. First, our findings display the different structural conditions that shape the wealth stratification process through homeownership for the current cohort of young adults’ cohort within and across countries. They reveal how homeownership inequality increases and decreases within and between countries depending on the extent of mortgage market liberalization and governmental housing policies, together with family financial support. The interaction between these mechanisms contributes to hierarchical patterns of homeownership among young adults in WE countries and to a more equalized distribution of housing asset ownership among SE and CEE countries.

Paradoxically, we found that when housing finance systems are more liberal (more generous and active), those receiving the highest amount of family financial support have a higher probability of mortgaged homeownership than those receiving lower amounts of family financial support. This counterintuitive finding is surprising, since one would assume that a more liberal mortgage market would reduce the advantages of the wealthiest in mortgaged homeownership. We offer two main explanations for it: First, countries with a more accessible mortgage market are usually also characterized by less affordable housing prices for the median-income household (see Table 1 above). This in turn makes young-adult households more dependent on their family financial resources [43,80], which provides an advantage for the wealthier in mortgage homeownership. Second, in those countries young adults may revert to family financial assistance in order to invest in housing assets in higher socioeconomic locations that will appreciate faster [83]. Therefore, liberal mortgage markets actually serve to enable wealthier young adults to reproduce and preserve their parental wealth status, just as they serve to widen wealth inequality between the older cohort and the present cohort of young adults [43].

From the latter finding, we can also learn about how residential segregation is developed in space. As housing cost inflation has been increasing substantially in most European countries in the last decade, with the COVID-19 pandemic fueling this development even further [119], combined with muted income growth, young adults are being segregated in the built environment, according to the means available to them from their own family and the mortgage market. Those receiving higher support purchase housing assets in higher socioeconomic areas that also provide higher quality of education and health services. The access of wealthier young adults to homeownership increases housing price inflation even more, thus “locking out” those who are less wealthy from access to the housing market as well. In this sense, the pursuit of wealth accumulation advances familistic solidarity over social solidarity. By contrast, when housing finance systems are more conservative, IWT gaps in homeownership still exist but to a much lower degree. We do not imply that conservative mortgage markets are a better alternative than liberal ones, but we do emphasize the need for alternative government housing programs in the rental and the housing market sectors to increase housing affordability among the young. The best example for this is the Netherlands, which is characterized by the most accessible mortgage market together with a highly controlled rental market, which reduces wealth inequality and wealth concentration to some degree.

Our research findings also hold demographical implications for the present cohort of young adults, specifically for those who cannot rely on family financial resources or receive them to a lesser extent.

Since the transition to first-time homeownership for young adults is usually coupled with marriage and family expansion, housing unaffordability also means a delay in the transition to first-time homeownership and in family formation, which can also translate to decreasing fertility rates at the national level [34].

While this study does make some novel contributions, it also has several limitations. It was not possible to follow the subjects over time, since no dataset is available on the existence and extent of IWT over time for the same households in different countries. Such information would provide further insight into the accumulation of wealth over individuals’ life cycles in different contexts. Future research would benefit from a larger sample of countries to examine how institutional factors moderate the association between *IWT* and housing tenure by means of multilevel models. In addition, since the HFCS survey does not collect data on NE countries (except for Finland), they are not represented in this study.

Lastly, as the sociological literature about wealth inequality continues to expand, the problem of housing unaffordability across young adults in differential social settings has to be more seriously confronted theoretically, empirically, and from a social policy perspective. Tackling the problem of housing unaffordability among young adults from the roots will require more sociological studies about the institutions that shape homeownership inequality in an area of intensified mortgage and housing financialization as well as increasing intra- and intergenerational wealth inequality.

## Supporting information

S1 Fig Percent of housing tenure, by country. (TIF)

S2 Fig Percent of housing tenure by gift receivers and country. (TIF)

S3 Fig Percent of housing assets receivers, by country. (TIF)

S1 Table (DOCX)

S2 Table (DOCX)

S3 Table (DOCX)

S4 Table (DOCX)
