# Supplementary figures and images for: Intergenerational wealth transmission and homeownership in Europe–a comparative perspective
Source: PLoS One. 2022 Sep 28;17(9):e0274647. doi: 10.1371/journal.pone.0274647 (PMC9518901; doi:10.1371/journal.pone.0274647)

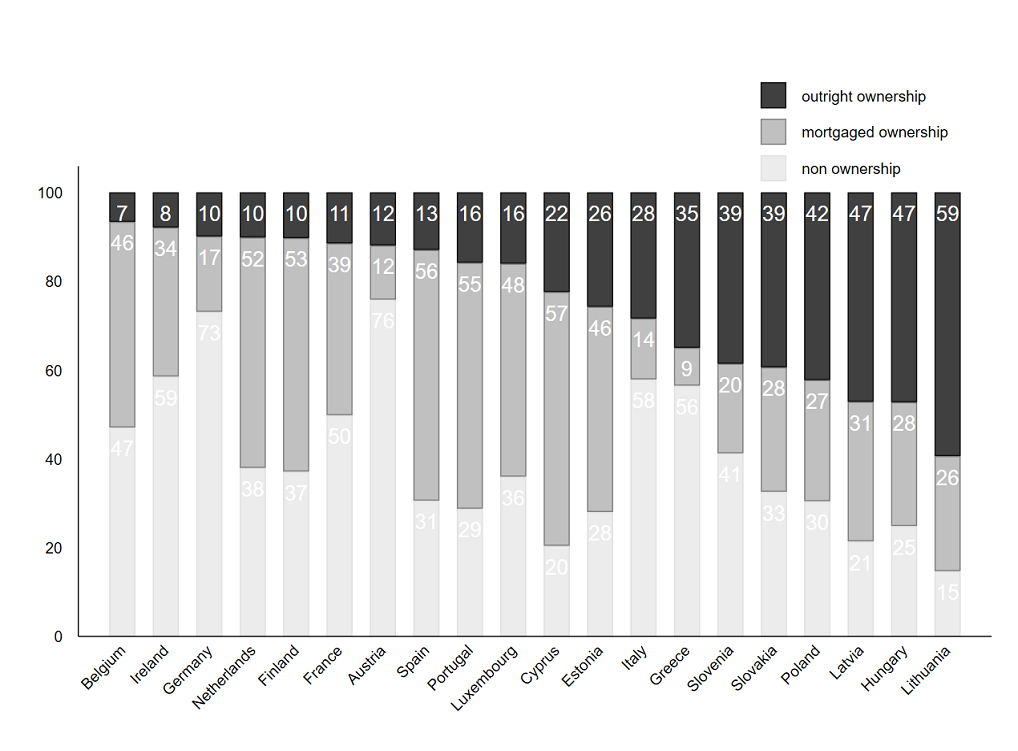

Supplement: S1 Fig — (TIF) [file pone.0274647.s001.tif]

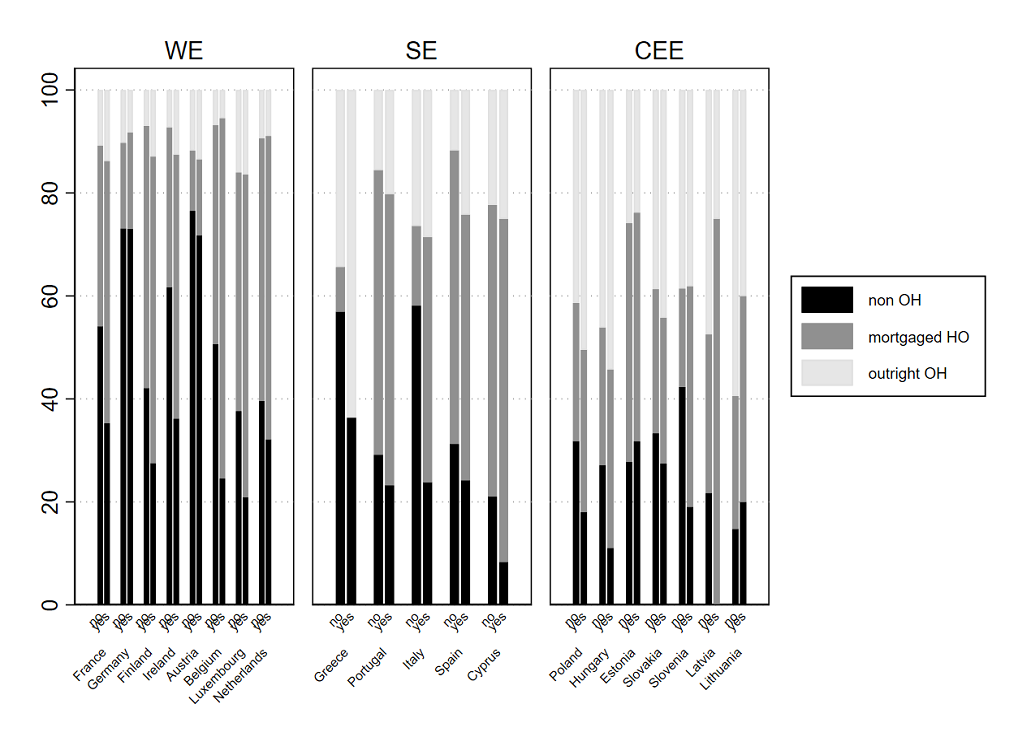

Supplement: S2 Fig — (TIF) [file pone.0274647.s002.tif]

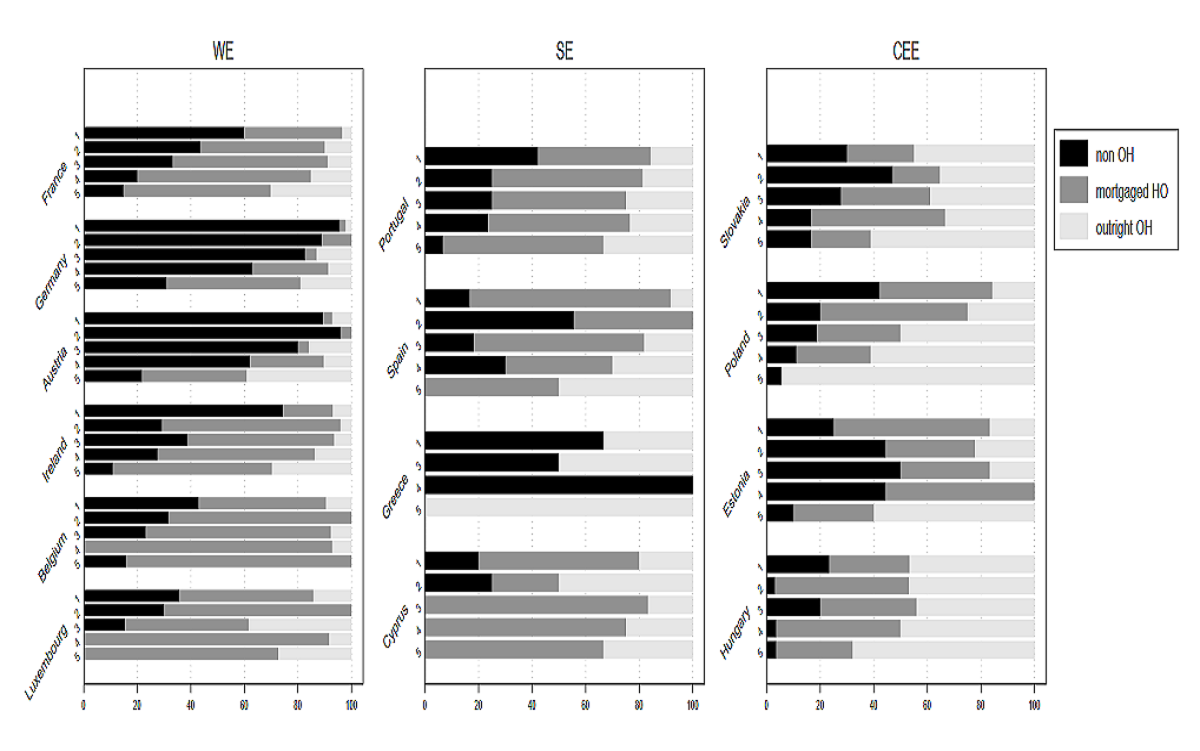

Supplement: S3 Fig — (TIF) [file pone.0274647.s003.tif]
