# Supplementary material for: Intergenerational wealth transmission and homeownership in Europe–a comparative perspective
Source: PLoS One. 2022 Sep 28;17(9):e0274647. doi: 10.1371/journal.pone.0274647 (PMC9518901; doi:10.1371/journal.pone.0274647)
Supplement: S1 Table — (DOCX) [file pone.0274647.s004.docx]

**Table A1. Relative risk Ratio from pooled multinomial logistic regression predicting the difference in probability of having outright ownership or mortgaged homeownership, by IWT, household socio-demographic variables and country level fixed effects (SE in parentheses).**

| **Variables** | **mortgaged HO** | **outright OH** |
| --- | --- | --- |
| *IWT* (received IWT=1) | 2.953*** | 8.581*** |
|  | (0.143) | (0.429) |
| *Age (*centered) | 1.108*** | 1.103*** |
|  | (0.00547) | (0.00633) |
| *Household income quintiles* (p2) | 1.736*** | 1.282*** |
|  | (0.109) | (0.0831) |
| (p3) | 2.976*** | 1.652*** |
|  | (0.186) | (0.111) |
| (p4) | 5.509*** | 2.266*** |
|  | (0.362) | (0.162) |
| (p5) | 8.417*** | 2.910*** |
|  | (0.599) | (0.228) |
| *Education level*; Secondary edu | 1.440*** | 1.494*** |
|  | (0.0886) | (0.105) |
| Tertiary education | 1.478*** | 1.565*** |
|  | (0.0956) | (0.118) |
| *Marital status;* married | 1.884*** | 1.238*** |
|  | (0.0874) | (0.0700) |
| divorced/widowed | 1.124 | 1.037 |
|  | (0.0841) | (0.0840) |
| Householdsize (centered) | 1.119*** | 1.118*** |
|  | (0.0191) | (0.0227) |
| Employment_status (1=employed) | 2.890*** | 1.418*** |
|  | (0.205) | (0.110) |
| Country level variables: |  |  |
| Postcommunist (1=yes) | 1.485 | 4.998*** |
|  | (0.302) | (0.995) |
| GDP percapita (centered) | 0.928*** | 0.958*** |
|  | (0.00817) | (0.00868) |
| Country#year dummies^a^ |  |  |
| Constant | 0.0306*** | 0.0601*** |
|  | (0.00429) | (0.00861) |
| Observations | 23,168 | 23,168 |
| Pseudo-R-squared | 0.261 | 0.261 |
| Log pseudolikelihood | -17954 | -17954 |
| chi2 Wald | 8404 | 8404 |

*** p<0.05, ** p<0.01, * p<0.001 Reference group; non-homeownership.

Omitted groups; had not received IWT, p1, elementary school, single, unemployed, not post- communist country. SE were calculated using bootstrap with 1,000 replications, weighted data.
a Not presented for the sake of brevity.

**Note.** Relative Risk Ratio is calculated by dividing the risk of the outcome (e.g. *mortgaged homeownership*)

for the people who were exposed to the risk (e.g. IWT beneficiaries) by the risk of the same outcome for the people who were not exposed to the risk (e.g. non-beneficiaries of IWT).

A risk ratio > 1 means increased risk of *mortgaged homeownership* for IWT beneficiaries.

A risk ratio < 1 means a reduced risk of *mortgaged homeownership* for IWT beneficiaries (For elaboration see; Hancock and Kent, 2016).
