# Supplementary material for: Intergenerational wealth transmission and homeownership in Europe–a comparative perspective
Source: PLoS One. 2022 Sep 28;17(9):e0274647. doi: 10.1371/journal.pone.0274647 (PMC9518901; doi:10.1371/journal.pone.0274647)
Supplement: S2 Table — (DOCX) [file pone.0274647.s005.docx]

**Table A2. Relative Risk Ratio from pooled multinomial logistic regression predicting the difference in probability of having outright ownership or mortgaged homeownership, by IWT quintiles, household socio-demographic variables and country level fixed effects (SE in parentheses).**

| **Variables** | **mortgaged HO** | **outright OH** |
| --- | --- | --- |
| IWT (p2) | 1.931*** | 2.997*** |
|  | (0.306) | (0.518) |
| IWT (p3) | 4.203*** | 5.650*** |
|  | (0.726) | (1.042) |
| IWT (p4) | 11.49*** | 18.76*** |
|  | (2.306) | (3.968) |
| IWT (p5) | 33.78*** | 68.66*** |
|  | (10.98) | (22.97) |
| **Hosehold level control variables:** |  |  |
| Age centered | 1.147*** | 1.126*** |
|  | (0.0190) | (0.0197) |
| hh income (p2) | 1.656*** | 1.216 |
|  | (0.344) | (0.241) |
| hh income (p3) | 1.986*** | 1.306 |
|  | (0.418) | (0.279) |
| hh income (p4) | 4.137*** | 1.818*** |
|  | (0.891) | (0.402) |
| hh income (p5) | 5.306*** | 1.783*** |
|  | (1.256) | (0.433) |
| Secondary edu | 1.525 | 1.180 |
|  | (0.374) | (0.296) |
| Tertiary education | 1.349 | 0.961 |
|  | (0.334) | (0.246) |
| married | 1.623*** | 1.055 |
|  | (0.258) | (0.185) |
| divorced/widowed | 0.696 | 0.699 |
|  | (0.203) | (0.202) |
| householdsize centered | 1.203*** | 1.132 |
|  | (0.0749) | (0.0750) |
| Employment_status (1=employed) | 3.601*** | 1.928*** |
|  | (0.872) | (0.447) |
| **Country level control variables:** |  |  |
| Postcommunist (1=yes) | 3.459*** | 12.67*** |
|  | (1.791) | (6.188) |
| GDP percapita centered | 0.950*** | 0.956*** |
|  | (0.0221) | (0.0218) |
| Country#year dummies a |  |  |
| Constant | 0.0452*** | 0.152*** |
|  | (0.0189) | (0.0614) |
| Observations | 3,302 | 3,302 |
| Pseudo-R-squared | 0.330 | 0.330 |
| Log pseudolikelihood | -2350 | -2350 |
| Chi2 Wald | 11260 | 11260 |

*** p<0.05, ** p<0.01, * p<0.001

Reference group; non-homeownership. Omitted groups; IWT (p1), hh income (p1), elementary school, single, unemployed, not post-communist country.
SE were calculated using bootstrap with 1,000 replications, weighted data.
a Not presented for the sake of brevity.
