# Supplementary material for: Intergenerational wealth transmission and homeownership in Europe–a comparative perspective
Source: PLoS One. 2022 Sep 28;17(9):e0274647. doi: 10.1371/journal.pone.0274647 (PMC9518901; doi:10.1371/journal.pone.0274647)
Supplement: S3 Table — (DOCX) [file pone.0274647.s006.docx]

**Table A3. Relative Risk Ratio from pooled multinomial logistic regression predicting the difference in probability of housing tenure by IWT and macro variables.**

*** p<0.05, ** p<0.01, * p<0.001 Reference group; non-homeownership.

Omitted groups; had not received IWT, household income (p1), elementary school, single, unemployed, not post- communist country, year 2010-2011.

Note. Country dummies are not included in the models due to perfect multicollinearity.
