# Supplementary material for: Intergenerational wealth transmission and homeownership in Europe–a comparative perspective
Source: PLoS One. 2022 Sep 28;17(9):e0274647. doi: 10.1371/journal.pone.0274647 (PMC9518901; doi:10.1371/journal.pone.0274647)
Supplement: S4 Table — (DOCX) [file pone.0274647.s007.docx]

**Table A4. Relative Risk Ratio from pooled multinomial logistic regression predicting the difference in probability of housing tenure by IWT quintiles and macro variables.**
